# Strategies Targeting Type 2 Inflammation: From Monoclonal Antibodies to JAK-Inhibitors

**DOI:** 10.3390/biomedicines9101497

**Published:** 2021-10-19

**Authors:** Andrea Matucci, Emanuele Vivarelli, Francesca Nencini, Enrico Maggi, Alessandra Vultaggio

**Affiliations:** 1 Immunoallergology Unit, Azienda Ospedaliero-Universitaria Careggi, 50134 Florence, Italy; emanuele.vivarelli@gmail.com (E.V.); francesca.nencini@unifi.it (F.N.); vultaggioalessandra@gmail.com (A.V.); 2 Immunology Department, Bambino Gesù Children’s Hospital, IRCCS, 00165 Rome, Italy

**Keywords:** asthma, Th2 cytokines, type 2 inflammation, monoclonal antibodies, JAK-inhibitors

## Abstract

Bronchial asthma and its frequent comorbidity chronic rhinosinusitis (CRS), are characterized by an inflammatory process at lower and upper respiratory tract, with a variability in terms of clinical presentations (phenotypes) and distinct underpin pathophysiological mechanisms (endotypes). Based on the characteristics of inflammation, bronchial asthma can be distinguished into type 2 (eosinophilic) or nontype 2 (noneosinophilic) endotypes. In type 2 asthma endotype, the pathogenic mechanism is sustained by an inflammatory process driven by Th2 cells, type 2 innate lymphoid cells (ILC2) and type 2 cytokines, which include interleukin (IL)-4, IL-5, IL-9 and IL-13. The definition of asthma and chronic rhinusinusitis phenotype/endotype is crucial, taking into account the availability of novel biologic agents, such as monoclonal antibodies targeting the classical type 2 cytokines. Recently, new therapeutic strategies have been proposed and analyzed in preliminary clinical trials. Among them Janus kinase (JAK) inhibitors, now largely used for the treatment of other chronic inflammatory diseases such as rheumatoid arthritis and inflammatory bowel diseases, is receiving great relevance. The rationale of this strategy derives from the data that JAK is a tyrosine kinase involved in the signaling of T cell receptor and of several cytokines that play a role in allergic respiratory disease, such as IL-2, IL-4 and IL-9. In this review, we discuss whether treatment with biological agents and JAK inhibitors may be equally effective in controlling type 2 inflammatory process in both asthma and CRS.

## 1. Introduction

Bronchial asthma (BA) and its frequent comorbidity, chronic rhinosinusitis (CRS), are characterized by an inflammatory process in the lower and upper respiratory tract, with variable clinical presentations (phenotypes) and distinct underlying pathophysiological mechanisms (endotypes). The latter have been defined as ”subtypes” of disease with a unique pathogenic mechanism; each endotype shows a functionally and pathologically different profile due to the involvement of specific molecules or cells. For this reason, according to the features of inflammation, BA and CRS can be distinguished into type 2 (eosinophilic) or nontype 2 (noneosinophilic) endotypes [1,2,3]. In type 2 asthma endotype, the pathogenic mechanism is sustained by an inflammatory process driven by type 2 T helper (Th2) cells, type 2 innate lymphoid cells (ILC2) and type 2 cytokines, which essentially include interleukin (IL)-4, IL-5, IL-9 and IL-13 [4]. Eosinophilic asthma prevalence is approximately 70% of all severe asthmatic cases [5]. Nontype 2 endotype asthma, also called T2 low asthma, is characterized by neutrophilic or paucigranulocytic airway inflammation, driven by cytokines such as IL-8, IL-17 and IL-22 [6,7,8]. CRS is often associated with BA, and the reason could be related to the shared genetic traits that have been observed by several authors; in fact, a study involving patients from European ancestry showed 38 genome-wide significant loci between asthma and allergic diseases, mainly belonging to skin, mucosal tissues and immune system [9]. CRS also shows remarkable heterogeneity at both phenotypic and endotypic levels. There is a general consensus on the presence of two major phenotypes, which were defined as subgroups of patients with homogeneous clinically observable characteristics based on nasal endoscopic findings: CRS with (CRSwNP) and without (CRSsNP) nasal polyps [10,11]. The simple partition into the classical Th1- and Th2-oriented diseases does not encompass the molecular heterogeneity observed in patients with CRS, and the clinical phenotype does not allow for adequately identifying the two distinct immunologic profiles. In fact, a cluster analysis indicates that CRS is not a homogeneous inflammatory disease and that endotypes are present with a wide variability of inflammatory profiles [3,12]. An analysis of tissue endotypes emphasizes the importance of IL-5, IL-13 and eotaxin in type 2 sinu-nasal inflammation [13]. The type 2 eosinophilic inflammation is found in about 80% of all patients with nasal polyposis, whereas CRSsNP, often characterized by type 1 or type 3 inflammation, is associated with the presence of neutrophils and elevated levels of IL-6, IL-8 and IL-17A [3,14,15]. Eosinophilic CRSwNP tend to be more severe than noneosinophilic ones with a higher trend of recurrence after surgery [16,17]. In a significant proportion of cases, both asthmatic and CRS patients can achieve disease control with the conventional treatment with local (inhaled) steroids. However, a proportion of them have poor or no control even with maximal medical therapy and surgery, the latter being a strategy largely used in the past for controlling the recurrence of polyposis. Monoclonal antibodies can be effective for patients with recalcitrant CRS, especially for patients that have CRS endotypes characterized by the dominance of specific cytokines. In fact, CRSwNP patients with a classical type 2 endotype are usually resistant to current therapies, exhibiting a high recurrence rate and, therefore, are considered to have difficult-to-treat CRS [18]. Biomarkers, such as absolute eosinophil count in peripheral blood and total and specific IgE, may be used as laboratory hallmarks of type 2 endotype, and can help in the decision to start a biologic therapy with monoclonal antibodies that are now available [19,20]. In fact, the definition of BA and CRS phenotype/endotype is crucial, taking into account the availability of novel biologic agents, such as anti-IgE, anti-IL-5/IL-5Rα and anti-IL4/IL-13Rα monoclonal antibodies, which can be prescribed to patients with poor or absent responsive to conventional asthma or CRSwNP treatments [21,22,23].

Janus kinases (JAKs) are signaling proteins associated with the intracellular domain of receptor subunits for multiple cytokines. JAKs regulate multiple biological processes including many aspects of both innate and adaptive immunity, hematopoiesis and cell proliferation. New therapeutic options became available last year for treatment of BA, CRSwNP and atopic dermatitis. Among them, Janus kinase (JAK) inhibitors, now largely used for the treatment of other chronic inflammatory diseases such as rheumatoid arthritis and inflammatory bowel diseases, are gaining great relevance [24,25]. The rationale of this strategy stems from the notion that JAK is a tyrosine kinase involved in the signaling of T cell receptor and of several cytokines that play a pivotal role in allergic respiratory disease, such as IL-2, IL-4 and IL-9 [24,25].

In this review, we discuss whether treatment with biological agents and JAK inhibitors may be equally effective in controlling type 2 inflammatory process in both asthma and CRS.

## 2. Type 2 Cytokines Signal Pathways

In the last few years, the pathogenic mechanisms which underline BA, CRSwNP and atopic dermatitis have been extensively reviewed and the new concept of type 2 inflammation introduced. Type 2 inflammation in the upper and lower airways and skin is characterized by the presence of key type 2 cytokines IL-4, IL-13 and IL-5. The endotype of type 2 inflammation is the result of a complex cellular network in which adaptive Th2- and innate cell responses represent two integrated systems in the production of IL-4, IL-5 and IL-13. To fully understand the biological effects of cytokines in the inflammatory process including type 2 inflammation, it is also important to analyze the pathways of intracellular signals that are activated after the interaction of a single cytokine with its specific receptor. The Janus kinase (JAK)–signal transducer and activator of transcription (STAT) pathway regulates multiple biological processes involving both innate and adaptive immune responses. In fact, many proinflammatory cytokines and growth factors are implicated in the pathogenesis of inflammatory diseases signal by the JAK–STAT pathways. JAKs regulate multiple biological processes including many aspects of innate and adaptive immunity, hematopoiesis and cell proliferation. Four JAKs have been described: JAK1, JAK2, JAK3 and TYK2. JAKs belong to the family of tyrosine kinases (TYKs), which consist of four structural domains composed of seven homologous regions named JH1–JH7 [26]. JH1 is the active catalytic phosphotransferase domain and the target of the JAK-inhibitors developed so far, which compete with adenosine triphosphate at the catalytic site [27,28]. Many cytokines involved in the pathogenesis of autoimmune and inflammatory diseases use JAKs and STATs to transduce intracellular signals. The STAT transcription factors transmit cytokine signals downstream from the JAKs. As already occurs in other chronic immune-mediated diseases such as rheumatoid arthritis and inflammatory bowel diseases, the intracellular signals represent targets for a new generation of drugs such as the so-called small molecule inhibitors. In fact, many JAK inhibitors, which also block STAT phosphorylation, have been developed for treating inflammatory diseases [29]. When a cytokine binds its JAK-associated receptor, it induces receptor dimerization and this allows JAK to phosphorylate the receptor’s cytoplasmic tails. The phosphorylated aminoacids in the receptor tails form a docking site for STAT family members, which are recruited to the receptor’s tails due to their SH2 domains. STATs are a family of cytoplasmic transcription factors that encompasses seven members (STAT1, STAT21, STAT3, STAT4, STAT5A, STAT5B and STAT6) that are phosphorylated by JAK and, after phosphorylation, can form dimers that are transferred to the nucleus where they regulate the expression of cytokine-responsive genes by combining to specific DNA elements [29,30]. Many asthma-relevant cytokines, including IL-4, IL-5 and IL-13, depend on JAK signaling to elicit an inflammatory response. Thymic stromal lymphopoietin (TSLP) is another JAK-dependent cytokine that plays an important role in type 2-high disease, serving as an alarmin upstream of TH2 cytokine production [31,32].

In asthmatic and CRS patients, the inflammatory process at bronchial and nasal level is a consequence of the effects of several factors, including cytokines as the classical type 2- and other cytokines as transforming growth factor (TGF)-β, IL-1 and IL-6 [33,34,35,36,37,38,39,40,41,42,43]. Type 2 cytokines exert their biological effects through the activation of different JAK pathways. The binding of IL-5 to its cell surface receptor, comprising α and β subunits, activates receptor associated JAKs, predominantly JAK2, which is critical for downstream signaling by STAT3 phosphorylation [31]. IL-4, which is a key pathogenic cytokine in allergic diseases, requires JAK1, JAK3 and complex interactions of STAT3, STAT5 and STAT6 [31]. STAT6 is implicated in Th2 and Th9 differentiation [41,43]. The importance of STAT6 in Th2 cell differentiation has been confirmed by the generation of STAT6-deficient mice showing lack of IL-4-induced Th2 response and IgE class switching in mice; these animals fail to mount Th2 responses either in vitro or in vivo [43]. IL-13 likewise binds IL-4Rα and can activate signaling through JAK1, but also binds a more specific receptor subunit, the IL-13Rα1 chain, which associates with the JAK-family kinase TYK2 [44]. IL-13 also binds a second receptor (IL-13Rα2) with higher affinity than IL-13Rα1. IL-13Rα2, in IL-13 signaling, has been considered merely as a decoy receptor that binds free IL-13 strongly, without eliciting signaling [45].

## 3. Monoclonal Antibodies Targeting Type 2 Inflammation

During the past decade, the development of biological agents targeting type 2 cytokines or their receptors represents a landmark advancement in the treatment of inflammatory diseases in which these factors play a central role, such as severe asthma and more recently CRSwNP and atopic dermatitis [4,5,19,46]. According to the International Severe Asthma Registry, type 2 asthma represents approximately 70% of severe asthma cases [4]. In eosinophilic asthma, eosinophils increase in the peripheral circulation and accumulate in the airway wall and lumen, causing mucus hypersecretion, bronchoconstriction and airway remodeling [47].

Given the association of IL-4, IL-5, IL-9, IL-13 and TSLP to asthma pathologies, all have been targeted with antibody-based therapeutics that bind either directly to the cytokines or their receptors. Biological agents targeting type 2 inflammation include anti-IgE (omalizumab), anti-IL-5 (mepolizumab and reslizumab), anti-IL-5Rα (benralizumab) and anti-IL-4/IL-13Rα (dupilumab) [48,49,50,51,52,53,54,55]. Presence of a specific type 2 endotype is assessed in clinical practice using blood eosinophil count, FENO, and total and specific IgE to identify the dominant driver (type 2 cytokines or IgE) [56]. Biological agents are more likely to be efficacious in patients with asthma, particularly those with higher FENO levels and blood eosinophils, atopic dermatitis, CRSwNP, allergies and OCS use. Omalizumab, a recombinant humanized IgG1 monoclonal antibody, selectively binds to free IgE molecules, independent from the specificity, blocking the binding site (Cε3 domain) for FcεRI, modulating and acting upstream of the IgE network and slowing or preventing both the early and late allergic inflammatory cascade [57]. The depletion of free IgEs induces a downregulation of FcεRI expression not only on mast cells and basophils, but also on dendritic cells, reducing their antigen presenting activity to T lymphocytes [58,59,60]. By interrupting the IgE-mediated inflammatory cascade at an early stage, thus reducing both early and late asthmatic responses, omalizumab improves exacerbations, lung function and asthma control, with greater effect on exacerbations demonstrated for patients with high FENO levels, circulating eosinophils and periostin [61,62,63,64]. The clinical use of omalizumab has been recently extended to the treatment of patients with refractory CRSwNP. Omalizumab, indeed, improves nasal polyp score (NPS), nasal congestion score (NCS) and sinu-nasal outcome test (SNOT)-22, and shows an overall good impact on patients’ quality of life (QoL) [22].

Mepolizumab, reslizumab and benralizumab are three mAbs that reduce eosinophilic inflammation and are recommended as add-on therapies for the treatment of patients with severe, uncontrolled asthma who exhibit an eosinophilic phenotype [65,66,67]. These biological agents have been designed taking into account the central role of IL-5 in the differentiation, maturation and survival of eosinophils [67,68,69]. While the effect of the anti-IL-5 mAbs has been related to their ability to indirectly target eosinophils, benralizumab, a humanized afucosylated mAb recognizing the α-subunit of the IL-5 receptor, exerts its effect directly by depleting eosinophils through antibody-dependent cell-mediated cytotoxicity (ADCC) [70,71].

Mepolizumab, by blocking the effect of IL-5, reduces exacerbation rates, improves lung function and reduces oral corticosteroid (OCS) exposure in severe asthmatic patients with a blood eosinophils count of ≥150 cells/μL. For mepolizumab, better clinical outcomes have also been observed in patients with a higher percentage of blood and sputum eosinophilia, with severe asthma forms associated with CRSwNP, and lower maintenance OCS requirement [49,72,73,74,75,76]. In fact, concerning CRSwNP, positive results in terms of improvement of nasal symptoms have been obtained in a recent clinical trial with mepolizumab [19]. As noted above, benralizumab, an anti-IL-5 receptor alpha (IL-5Rα) mAb, exerts the therapeutic effects by inducing a direct, rapid and nearly complete depletion of eosinophils via enhanced ADCC, providing enhanced clinical benefits for patients with late onset asthma, increased peripheral blood eosinophils, greater exacerbation history, poor lung function, OCS use and CRSwNP as comorbidity [45,50,51,70,71,72]. More recently, taking into account the complex but partial interplay between eosinophilic inflammation, remodeling and the role of the various type 2 cytokines, a deep attention has been dedicated to other type 2 cytokines as IL-4 and IL-13. Indeed, they have been clearly identified as preferential therapeutic targets since they play a central role in the pathogenesis of type 2 inflammation disorders, including BA, CRSwNP and atopic dermatitis [77,78,79,80]. In fact, dupilumab, a fully human mAb directed toward the α chain of IL-4 receptor used by both cytokines, has been recently introduced for treating all the type 2 related diseases. Dupilumab has been demonstrated to significantly reduce the rates of severe asthma exacerbations and OCS use and to improve lung function, effects particularly observed in patients with high peripheral blood eosinophils counts and FENO levels [49,78]. The beneficial effects are very pronounced in patients suffering from CRSwNP treated with dupilumab who display a rapid decrease of polyp size, radiological sinus opacification and symptoms severity [79]. Even though all the biologicals available exert significant clinical benefits in patients with type 2 diseases (asthma and/or CRSwNP and/or atopic dermatitis), clinical trials and real-life studies have highlighted a variable response to treatment [81]. In addition, while dupilumab is highly effective in controlling atopic dermatitis, no data are available regarding anti-IL-5/IL-5R monoclonal antibodies. We can hypothesize that, in atopic dermatitis, the axis IL-4-IL-13 is crucial in the pathogenesis of skin inflammation while IL-5/eosinophyl pathway is dispensable.

Airway epithelial cells represent the first line of defense in the mucosal surfaces. In response to injury due to allergens and pathogens, airway epithelial cells secrete cytokines such as IL-25, IL-33, TSLP and granulocyte macrophage colony stimulating factor (GM-CSF) [82]. These cytokines can activate dendritic cells and ILC, promoting production of Th2 cytokines and provoking a T2 high inflammation [82]. These cytokines have therefore been studied as useful targets for BA. Indeed, tezepelumab, a fully human anti-TSLP monoclonal antibody, has been shown to improve asthma control and FEV1 and reduce exacerbations, blood eosinophyl count, FENO and total serum IgE in phase II trials [83,84]. An anti-IL-33 monocloclonal antibody, REGN3500, has been shown to prevent airway remodeling in a murine model of house dust-mite-induced asthma and to reduce eosinophyls infiltration and airway hyperreactivity in an ovalbumin induced asthma murine model [85,86,87]. IL-25 blockade has also displayed promising results in murine models of ovalbumin-induced and house dust-mite-induced asthma, but clinical trials on humans are still awaited [88,89].

Another attractive target for asthma therapy was IL-13, which has an important role in goblet cells hyperplasia and airway remodeling [90]. Unfortunately, several monoclonal antibodies targeting IL-13 (lebrikizumab, tralokinumab, GSK679586) were not able to reduce exacerbations in clinical phase-2 and phase-3 trials [91,92,93]. Lastly, anti-IL9 monoclonal antibody, MEDI-528, which was developed considering the role of IL-9 in mast cell biology, also failed to show efficacy in phase-2 trials [94].

The existence of a range of response among biologicals is likely due to differences in target and patients’ baseline features. After all, during the type 2 inflammatory process, the role of the individual cytokines is not exclusive and each can participate to a greater or lesser extent in the initiation, maintenance and amplification of the inflammatory process [95]. Furthermore, the role of individual cytokines and of the cells they affect may be different at the individual level, in the different phases of the disease and in the different tissues (bronchial and nasal mucosa, skin). In addition, histological alterations of the airways wall in asthma and CRS change over time at least in partly because of the effect of treatment [95]. For this reason, despite the therapeutic success of biologics, it has become evident that targeting a single cytokine does not completely abrogate the type 2 disorder in the great majority of patients.

## 4. JAK-Inhibitors Targeting Type 2 Cytokine Pathways

Although the focus of JAK inhibitors for the treatment of chronic inflammatory conditions has been on RA and IBD, there are other conditions in which JAK inhibitors could serve as therapeutic options. In fact, also taking into account the role of the JAK pathways in the transmission of intracellular signals of type 2 cytokines involved in asthma pathogenesis, new therapeutic strategies interfering with JAK appear at the horizon for treating patients suffering from the severe form of bronchial asthma, CRS and atopic dermatitis. Actually, many JAK inhibitors, which also inhibit STAT phosphorylation, have been developed for treating inflammatory diseases [24,25]. Tofacitinib is a potent pan-JAK antagonist that is notably more selective against JAK1 and JAK3, which are critical in Th2 signaling, than against JAK2 and TYK2 [96]. Experimental data obtained in mouse model of pulmonary eosinophilia, show that systemic administration of the JAK3 inhibitor tofacitinib (CP-690550), approved for the treatment of rheumatoid arthritis, ulcerative colitis and moderate-to-severe chronic plaque psoriasis [97,98,99,100,101,102,103], effectively inhibited antigen-induced pulmonary eosinophil influx, IL-13 and eotaxin expression [99]. These data are consistent with the previous demonstration that JAK3^−/−^ mouse models failed to exhibit efficient recruitment of Th2 cells to the lungs following antigen challenge [104]. In addition, by considering that IL-9 uses the JAK3 pathway for the signal transmission and has also been implicated in the development of allergic pulmonary inflammation, the effects of JAK3 block could be related to the downstream inhibition signal [13,31]. More recently, topical tofacitinib effectively reduced overall inflammation in a murine model of CRSwNP by suppressing Th2-dominant inflammation [105]. The possible use of topical JAK inhibitors has also been evaluated in asthma patients. In fact, in adult subjects with mild asthma and FENO higher than 40 parts per billion (ppb), inhaled tofacitinib induced dose-dependent reduction of FENO [106]. In the group of type 2 diseases, JAK inhibitors have been largely evaluated in patients suffering from atopic dermatitis. In moderate-to-severe atopic dermatitis, a small open-label clinical trial with oral tofacitinib in six patients who were refractory to standard treatment showed a decrease in SCORAD from 36.5 at week 8 to 12.2 at week 29, with no adverse events [106]. More importantly, 2% topical tofacitinib in mild-to-moderate atopic dermatitis patients showed a significantly decrease of EASI score compared with the control group (–81.7% vs. −29.9%) [99]. In addition, baricitinib, an oral small molecule with potent JAK1 and JAK2 antagonism, has been demonstrated to be effective in reducing the skin lesions and pruritus in atopic dermatitis patients and improving HRQoL [107]). The JAK1 inhibitor abrocitinib, which reduces IL-4 and IL-13 signaling, is being investigated for the treatment of atopic dermatitis. The 200 mg dose of abrocitinib was superior to dupilumab with respect to itch response at week 2 but not with respect to most other key clinical features of the disease [108]. Taking this into account, it could be hypothesized that JAK inhibition in type 2-low asthma endotype may also be effective in a proportion of asthmatic patients; the endotype is represented by the type 2-low pattern. This observation seems to suggest that tezepelumab, a monoclonal anti-TSLP antibody being studied in patients with asthma, proves to be effective regardless of type 2 endotype in producing a consistent reduction in asthma exacerbations, considering that TSLP exerts its biological effects through the JAK1/JAK2 pathway [83]. One of the major concerns during treatment with biological agents has been related to the safety profile. Data obtained from the clinical trials and in real-life studies have been clearly confirmed not only the efficacy but also the safety of the biological agents targeting type 2 cytokines [109]. In addition, the high safety profile is also confirmed by the authorization to administer omalizumab, the first biological agent for treating severe asthma, in pregnant women [110]. To date, the safety profile of JAK inhibitors represents a key point of discussion specifically when these drug are used systemically for the treatment of immune-mediated diseases such as asthma and atopic dermatitis. Most of the safety data come from the large trials of in rheumatic and inflammatory bowel diseases. In recently published analysis using data from long-term extension studies in patients suffering from rheumatoid arthritis and treated with tofacitinib, emerges a major incidence for severe infections but similar to those observed biologics as anti-TNF-α monoclonal antibodies, currently used in clinical practice for the treatment of this disease, as noted in [111]. However, an increase of the risk of herpes zoster infection compared with biologics is observed [112]. Cardiovascular risk was one of the concerns raised about JAK inhibitors, largely related to the alterations in lipid profile noted with this class of drugs [113]. To avoid rapid absorption across the lung and into systemic circulation, design of lung-restricted molecules must include a lung retention strategy. To maximize this objective, inhaled design must contemplate not only retention of the compound within the lung, but also access of the drug to the relevant biological target. In addition, to reduce the possible negative impact of a systemic treatment, a selective potent inhaled JAK inhibitor (GDC-0214) has been used in patients with mild asthma. The biological effect caused dose-dependent reductions in FENO for mild asthma. There were no major imbalances in adverse events or laboratory findings, or evidence of systemic JAK inhibition. As the authors mentioned, however, subsequent trials will be needed to confirm whether or not the observed reduction in FENO will translate to improvements in airflow obstruction, symptoms and exacerbations among populations with a broader spectrum of asthma severity [26].

## 5. Conclusions

The definition of the pathogenic mechanisms underpinning type 2 diseases such as BA, CRS and atopic dermatitis has led to the design of new therapeutic strategies based on the use of biological drugs targeting specific cytokines and cytokine receptors. The use of biologics directed toward specific pathways of inflammation has improved the efficacy of asthma control [19]. Severe asthma represents the best model to understand the efficacy of biological agents in achieving the clinical control of the disease. In fact, biologics are highly effective in reducing exacerbations, diminishing symptoms and improving lung function in well-defined asthma populations. The biological agents have also been recently introduced in the treatment of CRSwNP and atopic dermatitis, in which type 2 inflammation represents the pathological background, at least in a proportion of patients [21,22,46].

The efficacy of biological agents clearly demonstrated in the clinical trials and real-life studies allows us to consider these agents as the “cornerstone” for treating type 2 diseases. However, in some patients, biologics achieve some but not all criteria for a remission of the disease [81]. If we consider asthma as a model, disease control includes absence of symptoms, optimization and stabilization of lung function, and absence of the use of systemic corticosteroids that is obtained in a high percentage but not in all patients [81]. For these reasons, in patients with uncontrolled disease, identification of a specific endotype is crucial in clinical practice to guide therapy, not only at the beginning of treatment but also during the maintenance phase of treatment. As already mentioned, currently, the available biological agents for treating type 2 diseases are represented by monoclonal antibodies targeting the well-known type 2 cytokines (IL-4, IL-5 and IL-13) or their receptors. The pharmacological research is deeply involved not only in the realization of new monoclonal antibodies but also of other drugs. In fact, at the horizon we can also see new therapeutic strategies based on the use of JAK inhibitors for type 2 diseases. Topic JAK inhibitors offer an intriguing new class of treatments for asthma, CRSwNP and atopic dermatitis, all included in the group of type2 diseases. Several important questions remain to be clarified, specifically concerning the safety profile. The administration of an inhaled JAK inhibitor to patients with asthma might reduce the inflammatory process while minimizing systemic exposure and any associated toxicities. This is also valid for the other type 2 diseases such as CRS and atopic dermatitis. Given the wide range of effector molecules that use the JAK/STAT pathways, the latter is becoming an increasingly attractive therapeutic target for a wide range of immune-mediated diseases beyond rheumatic and inflammatory bowel diseases. In fact, currently, JAK inhibitors have been approved for the treatment of immunomediated diseases such as rheumatoid arthritis, psoriasis and inflammatory bowel diseases. JAK inhibitors appear to have a potential position in the treatment of atopic dermatitis, BA and CRS. The rationale of efficacy is the direct effects of the block of the mechanisms of signal transduction downstream of the cytokine receptors involved in the pathogenesis of type 2 diseases. Taking into account the central role of the JAK pathways in the physiology of several cell types, the acceptable safety profile of JAK inhibitors remains a mandatory aim, particularly in clinical condition such as type 2 diseases. It is important to remember that JAK inhibitors not only block type 2 cytokines, but also influence type 1 cytokines, which are deeply involved in the defense toward infectious agents [114]. It is highly likely that potent pan-JAK inhibition will provide maximal efficacy in the majority of patients, however, and with the greatest risk of toxicity due to broad pathway suppression. To date, however, the safety profile of JAK inhibitors appears to be acceptable for the treatment of these immune-mediated diseases. Although JAK-inhibition strategy offers a potential novel therapy that targets multiple cytokines involved in type 2 inflammation, the definitive clinical role in these diseases needs to be defined. Certainly, the already available monoclonal antibodies targeting the type 2 cytokines, its receptors and IgE have demonstrated high efficacy in addition to long-term safety.
